# Supplementary material for: Sleep Medication in Older Adults: Identifying the Need for Support by a Community Pharmacist
Source: Healthcare (Basel). 2022 Jan 13;10(1):147. doi: 10.3390/healthcare10010147 (PMC8775744; doi:10.3390/healthcare10010147)
Supplement: Supplementary file 1 [file healthcare-10-00147-s001.zip › healthcare-1476982-supplementary.pdf]

## Supplementary Material

**Sleep medications & hypnotics**

Patient's details

*(to be prepared before the interview and then anonymized (patient's initials only) in the internship report)*

First (given) name, family name: Age:

First (given) name, family name of the patient's family physician:

Weight: kg Height: m

Zip code for place of residence:

Does the patient have a personal prescription history file managed by the French national health insurance fund: ☐ Yes ☐ No (if "no", try to convince the patient to open one)

Recently discharged from hospital: ☐ Yes ☐ No

Medical history

☐ Memory disorder ☐ Cardiovascular disease (high blood pressure, chronic heart failure …)

☐ Dyslipidemia ☐ Urinary disorder (urinary incontinence, pollakiuria, urinary urgency…)

☐ Diabetes ☐ Chronic kidney disease

☐ Neurological disease (stroke, Parkinson's disease…) ☐ Dysthyroidism

☐ Respiratory insufficiency ☐ Other:

Current medications (number of prescription drugs: … number of OTC drugs: …)

Indicate drugs (trade name, INN, and dosage):

Highlight the medication(s) that you [the interviewer] think has/have been prescribed for sleep disorders:

- -
-

Points of concern regarding ongoing medications: ☐ Yes ☐ No
Please specify:

Date of the first interview:

Dates of the following prescription fulfillments

**Analysis of the patient's medical records**

*To be performed before the interview*

## Prescription history

Medication(s) taken for sleep disorders, and the prescribed dose level:

| Trade name and international nonproprietary name | Current dose level | Earliest dispensing date found | Ongoing/discontinued (specify discontinuation date) |
|---|---|---|---|

| | | | |
|---|---|---|---|
| | | | |
| | | | |
| | | | |
| | | | |

Have you found any overlaps between prescriptions of different hypnotic medications?
☐ Yes ☐ No

Does the medication's dosage comply with the summary of product characteristics? ☐ Yes ☐ No

Has the sleeping medication's dose level been
☐ increased in the last 6 months?
☐ decreased in the last 6 months?

Did you find any other prescriptions of central nervous system psychotropic drugs/depressants in the patient's records?
☐ Yes ☐ No
If so, which drugs? -
-
-
-

**Interview with the patient: ☐ alone ☐ accompanied**

**Information given by the patient during the interview**

## Tell me about yourself

Do you live alone? ☐ Yes ☐ No

What is/was your job?
Retired ☐ Yes ☐ No

What is/was your spouse's occupation?
Retired ☐ Yes ☐ No

Did you have non-standard working hours? ☐ Yes ☐ No
☐ daytime ☐ nighttime ☐ part-time

Do you do regular physical activity? ☐ Yes ☐ No

Do you get out of the house every day? ☐ Yes ☐ No

Do you take a nap? ☐ Yes ☐ No
If so, how long does it last?

Do you smoke? ☐ Yes ☐ No
If so, do you smoke in the evening? ☐ Yes ☐ No

Do you have any worries at the moment? ☐ Yes ☐ No
If so, could you indicate the level of worry on a scale of 1 (least) to 10 (most)?

Have you fallen over in the last 6 months? ☐ Yes ☐ No
If so, how many times?

## Tell me about your sleep

Do you go to bed at the same time every day? ☐ Yes ☐ No
If not, why not? ☐ I wait until I feel sleepy
☐ I do activities some evenings
☐ Other:

At what time do you go to bed?

How long does it take you to fall asleep?

Do you have difficulty falling asleep? ☐ Yes ☐ No
If you have difficulty falling asleep, what do you do?
*Reading, snacking, taking medication, watching television, staying in bed, turning on the light …*

Do you wake up at night? ☐ Yes ☐ No
If so, how many times a night?
If so, why do you wake up? ☐ disturbed by your spouse
*(Check all that apply)* ☐ noise in the house
☐ disturbed by an animal

☐ noise outside
☐ snoring
☐ nightmare
☐ acid reflux (gastroesophageal reflux disease)
☐ going to the toilet (nocturia)
☐ problems breathing, nighttime cough
☐ hot flushes
☐ stress/anxiety
☐ pain
☐ other

If you wake up at night, what do you do?
*Reading, snacking, taking medication, watching television, staying in bed, turning on the light, etc.....?*

At what time do you wake up?

At what time do you get out of bed?

Do you feel refreshed when you wake up? ☐ Yes ☐ No

Do you have problems concentrating or problems with your memory? ☐ Yes ☐ No

## Before bedtime

Tell me about your evening meal
Notes: a large evening meal ☐ Yes ☐ No
Do you drink alcohol (wine, beer, etc.) with the meal? ☐ Yes ☐ No
Do you drink coffee or tea after the meal ☐ Yes ☐ No

Do you do any activities in the evening? ☐ Yes ☐ No
If so, which ones?
Do you sometimes fall asleep during this activity? ☐ Yes ☐ No

Do you have a "ritual" before going to bed? ☐ Yes ☐ No
If so, please specify: ☐ drinking herbal tea
☐ taking a hot bath or shower
☐ reading
☐ taking sleep medication
☐ other

## Your bedroom

Are you sometimes bothered by light and/or noise? ☐ Yes ☐ No
*Option: ask whether any pets are present, or whether the neighborhood is noisy, etc.*

Do you watch TV in bed? ☐ Yes ☐ No

Do you use a computer/tablet/mobile phone in bed? ☐ Yes ☐ No

Can you see your alarm clock from your bed? ☐ Yes ☐ No

What is the temperature in your bedroom at night?

**Your sleep medications**

What medications are you taking to help you sleep?
*(Don't influence the patient - don't change their answers.)*

Apart from the sleep medication, are you taking any other medications? ☐ Yes ☐ No
If so, which ones?

How long have you been taking your sleep medication?

When was it first prescribed?

Was it first prescribed when you were in hospital? ☐ Yes ☐ No

Have you always taken the same sleep medication? ☐ Yes ☐ No
If not, which sleep medication did you take before?
Have you always taken the same dose? ☐ Yes ☐ No

At what time do you take the medication?

Do you sometimes change the dose yourself? ☐ Yes ☐ No
*(Try to find out here whether the patient has attempted or wishes to stop)*

Do you ever take another dose during in the night? ☐ Yes ☐ No

Have you ever run out of medication? ☐ Yes ☐ No
If so, how did you deal with this situation?

Do you manage your medications yourself? ☐ Yes ☐ No

Would you like me to come to your home to review your medicine cabinet and the management of your medications? ☐ Yes ☐ No
*(If yes, make an appointment with the patient, if the course supervisor agrees)*

### *Patients on benzodiazepines: the ECAB questionnaire*

Could you please take a few moments to fill out this questionnaire?
I can fill it out for you, if you wish.

*Administer the ECAB questionnaire* (Echelle Cognitive d'Attachement aux Benzodiazépines, *Cognitive Scale for Benzodiazepine Attachment). The ECAB questionnaire consists of 10 items that are scored as 1 or 0. Award 1 point if the answer is "true" (except for question 10, where 1 point is awarded if the answer is "false"). The total score for the questionnaire is obtained by adding up the points from each item. A score of 6 or more corresponds to benzodiazepine dependency with a sensitivity of 94% and a specificity of 81%.*

| | True | False |
|---|---|---|
| 1. Wherever I go, I need to have this medication with me | ☐ | ☐ |
| 2. This medication is like an addictive drug for me | ☐ | ☐ |
| 3. I often think that I will never be able to stop taking this medication. | ☐ | ☐ |
| 4. I avoid telling my family and friends that I am taking this medication. | ☐ | ☐ |

| | | |
|---|---|---|
| 5. I feel like I am taking too much of this medication. | ☐ | ☐ |
| 6. Sometimes, I'm afraid I might run out of this medication. | ☐ | ☐ |
| 7. When I stop taking this medication, I feel very sick. | ☐ | ☐ |
| 8. I am taking this medication because I can't do without it anymore. | ☐ | ☐ |
| 9. I am taking this medication because I feel bad when I stop. | ☐ | ☐ |
| 10. I only take this medicine when I need it. | ☐ | ☐ |

*Indicate the result here after retrieving the questionnaire:*

## Consider the following

Have you ever wanted to stop taking sleep medication? ☐ Yes ☐ No

If so, what do you intend to do now?

If so, why did you want to stop taking it?

If so, how did you go about stopping?

How and why were you prompted to start taking the sleep medication?

Would you like to stop taking the medication? ☐ Yes ☐ No
(Do you feel able to stop? Are you keen to stop?)

If so, can I contact your family physician?

## SUMMARY OF KEY POINTS DURING THE INTERVIEW

*If there are any key points, note the advice given to the patient and, if necessary, summarize the key points to be raised with the family physician (to be validated by the internship supervisor before contacting the physician).*

## CONTACT WITH THE FAMILY PHYSICIAN

Contact with the family physician? ☐ Yes ☐ No
*Specify the interview's impact on the patient's care.*
